# Percutaneous Coronary Interventions in Women

**DOI:** 10.1007/s11883-023-01150-x

**Published:** 2023-10-10

**Authors:** Golsa Joodi, Sristi Palimar, Marcella Calfon Press

**Affiliations:** 1 Department of Medicine, Division of Cardiology, David Geffen School of Medicine at UCLA, Los Angeles, CA USA; 2 Institute for Society and Genetics, UCLA, Los Angeles, CA USA

**Keywords:** Coronary artery disease, Percutaneous coronary intervention, Women, Outcomes

## Abstract

**Purposeof Review:**

Cardiovascular disease is the leading cause of morbidity and mortality among women globally. Numerous studies show ongoing disparities in diagnosis, management, and outcomes of ischemic heart disease in women compared to men. We aim to review the factors contributing to sex-based differential outcomes of percutaneous coronary interventions in women.

**Recent Findings:**

Hormonal influence on coronary arteries and progression of atherosclerosis in women results in distinct coronary plaque characteristics and unique pathological process such as spontaneous coronary artery dissection and myocardial infarction with non-obstructive coronary arteries. During the presentation of acute coronary syndromes, women are older and have higher burden of comorbidities, with higher short- and long-term mortality.

**Summary:**

Awareness of differences in vascular biology and unique risk factors for cardiovascular disease in women is essential for sustained improvement in cardiovascular mortality. Better representation of women in trials is crucial to address the gaps in knowledge and allow for individualized treatment approaches in women.

## Introduction

Cardiovascular disease (CVD) is a leading cause of morbidity and mortality among women in the USA and globally [1, 2]. This recognition has led to numerous statements and campaigns from professional societies highlighting the gaps in diagnosis and treatment of CVD among women [3••]. According to Global Burden of Disease report, the age-standardized CVD death rates have declined among women from 1995 to 2015, although this favorable trend was mostly seen in higher-income countries [4]. More recently, the rate of decline has slowed considerably, with an alarming increase in the rates in some developed countries [5]. Ischemic heart disease (IHD) accounts for majority of CVD and is the main driver of worsening CVD mortality trend seen in women [6•, 7] as evidenced by rising number of acute myocardial infarction (AMI) among women in the past decade [8, 9]. Despite significant scientific and technological advancements in medical and percutaneous treatment options, men and women demonstrate disparate treatment outcomes across adverse cardiovascular conditions such as acute coronary syndrome (ACS) and coronary artery disease (CAD). This disparity disproportionately undermines women’s cardiovascular health and can be attributed to atypical presentation of cardiovascular pathology, later diagnosis and treatment, and limited awareness among marginalized female demographics. Additionally, major sex differences exist in the pathophysiology and psychosocial factors associated with CVD. These issues are exacerbated by the under-representation of women in majority of outcomes trials, reinforcing the challenge to adequately assess cardiovascular health and implement effective treatment strategies in women. In this review, we aim to provide a summary of factors contributing to sex-based differential outcomes for percutaneous coronary intervention (PCI) by highlighting the differences in pathophysiology, presentation, and management of ACS in women.

## Risk Factors for CAD in Women

Traditional risk factors for CAD such as age, smoking, obesity, hypertension, hyperlipidemia, and diabetes mellitus are well established. It is important to note, however, that the risk conferred by these factors varies by gender, and they are associated with higher relative risk of CAD in women compared to men [10, 11]. Women of ethnic and racial minorities experience disproportionately higher prevalence of these risk factors [7]. In addition, due to hormonal influence on vascular biology, women are older than men at the time of diagnosis and are more likely to have clustering of concomitant risk factors and comorbidities. Other risk factors include comorbid conditions that lead to acceleration of atherosclerosis and such as chronic kidney disease, inflammatory disorders, autoimmune diseases, connective tissue disorders, and chronic infection. Common psychosocial disorders such as anxiety and depression are associated with a 22% increase in risk of developing IHD [12].

In addition to these risk factors that affect both men and women, there are several female-specific risk factors which significantly enhance future risk of CVD. These include age at onset of menarche and menopause, total reproductive years, polycystic ovary syndrome, infertility, spontaneous pregnancy loss, and adverse pregnancy outcomes (APO) such as hypertensive disorders of pregnancy, gestational diabetes, and preterm delivery [13]. APOs are thought to originate from defective placentation and share similar underlying metabolic and vascular abnormalities with CVD. Cardiac structural and functional remodeling such as left ventricular hypertrophy and diastolic dysfunction are seen in pregnancies complicated with APOs [14, 15]. Women with prior APO were found to have lower coronary flow reserve indicative of microvascular dysfunction in women with ischemic symptoms but no obstructive CAD [16]. Stable CAD is primarily managed by lifestyle and risk factor modification and optimal medical therapies. PCI is most beneficial in patients who present with acute coronary syndromes. The remainder of this review will focus on gender-specific differences in ACS and PCI.

## Pathophysiology of ACS in Women

Acute coronary syndromes are thought to be caused by acute rupture of lipid-laden plaque followed by platelet rich thrombosis in approximately 64% of patients [17]. An alternative mechanism involves plaque erosion with formation of thrombus on fibrointimal plaque which accounts for up to 25% of ACS, and was found to be more common in women than men (37.4% vs 18.5%) in autopsy studies [18]. Additional mechanisms of ACS include coronary spasm, spontaneous coronary artery dissection (SCAD) and other causes of myocardial infarction with nonobstructive coronary arteries (MINOCA).

SCAD is an increasingly recognized cause of AMI among young and middle-aged women who otherwise have few or no traditional CVD risk factors. More than 90% of SCAD events are in women, and it may be the cause of ACS in up to 35% of AMIs in women younger than 50 [19] and primary cause of pregnancy-associated MI. While the exact etiology is unknown, but there is an association with systemic arteriopathies such as fibromuscular dysplasia and extra-coronary aneurysms or dissections. Notably, diagnosis of SCAD and coronary interventions are much more challenging and associated with higher risk due to increased risk of iatrogenic dissection and other complications [19].

MINOCA is defined as the presence of AMI criteria in the setting of non-obstructive CAD (less than 50% angiographic stenosis). MINOCA is more common in women (40–60%), as opposed to men who are more likely to have obstructive CAD. Proposed mechanisms include coronary microvascular dysfunction, altered endothelial tone and response to vasodilator stimuli. MINOCA patients are less likely to receive secondary prevention medications and have an approximately 40% increase in all-cause mortality compared to patients with non-obstructive CAD with stable angina [20].

The most common presentation of ACS chest discomfort is seen in 79% and 74% of men and women. Although previously women were thought to have “atypical” symptoms, it is now recognized that sex differences may be due to differences in interpretation and description of symptoms [21]. However, women are more likely to describe other symptoms such as dyspnea, neck pain, left arm and shoulder pain, palpitations, jaw pain, nausea or vomiting, indigestion or epigastric pain [21]. Younger women are more likely to present without chest pain, and 1 in 5 women perceive their symptoms as being related to anxiety or stress [22]. Due to these perceptions, women commonly present later at the time of ACS, which in turn leads to delays in revascularization from symptom onset associated with worse outcomes.

## Treatments and Outcomes in Women Presenting with ACS

In acute coronary syndromes, women are less likely to receive reperfusion or to undergo PCI [23]. Thrombolytic therapy, when administered early, reduces mortality and is recommended per guidelines in patients without contraindications who present to a non-PCI capable center with an anticipated delay to PCI of > 120 min of first medical contact. Despite this, women less frequently receive thrombolytics and are more likely to have fatal and non-fatal complications [24], with female sex identified as an independent risk factor for intracranial hemorrhage [25] or major bleeding [26]. Primary PCI is the cornerstone of management of ACS and in particular STEMI. In large, multi-institutional observational study assessing temporal trends and sex differences in revascularization strategies among young patients (18–59) presenting with STEMI from 2004 to 2011, women were less likely to undergo PCI, although over the duration of study there was significant improvement in the rates [23]. Similarly, in Harmonizing Outcomes with Revascularization and Stents in Acute Myocardial Infarction (HORIZONS-AMI) trial, women were more often treated with medical management alone (i.e., lower rates of PCI) and had significantly higher rates of 3-year major adverse cardiac events (MACE) and major bleeding [26]. A retrospective analysis of over 100,000 coronary angiograms in Sweden and Iceland from 2007 to 2011 showed that women were less likely to undergo PCI following the diagnosis of 1-vessel disease. Among patients with 2- or 3-vessel or left main involvement, women were more likely to undergo PCI and less likely to be referred for CABG [27].

Regarding long term outcomes, in a large-scale pooled analysis of 23 contemporary PCI trials, (*N* = 32,877, 27.8% women) undergoing PCI with early and new-generation drug eluting stents (DES), women had higher rates of composite outcome of death, MI, or ischemia driven target lesion revascularization, all-cause death, and cardiac death and ischemia-driven TLR after 5 years of follow-up [28•]. Similar results showing worse short- and long-term morbidity and mortality following PCI in women have been redemonstrated in many trials and observational studies.

This may be in part due to older age and more prevalent comorbidities in women compared to men at the time of diagnosis. However, our evolving understanding of differences in pathophysiological processes and vascular biology in women suggests that these factors directly influence the clinical outcomes.

ST-elevation MI (STEMI) and non-ST-elevation MI (NSTEMI) account for approximately 30% and 70% or ACS in women [17). While overall there was a reduction in all types AMI among women older than 65 in the French nationwide study 2004–2014, younger women experienced 6.3% increase in ACS rate [9] driven by increase in STEMI (+ 21.7%) and NSTEMI (+ 53.7%). Chronic coronary syndrome and stable ischemic heart disease with angina are the other common indications for PCI.

### ACS/STEMI

Despite known mortality benefit for primary PCI in the setting of STEMI, several US and international studies have repeatedly demonstrated lower rates of provision of invasive management in women [9, 23, 29]. Although these disparities may be to some extent explained by differences in age and comorbidities, differences persist after adjustment for these differences.

### ACS/NSTEMI

Early invasive strategy in patients with non-ST-elevation myocardial infarction (NSTEMI) is associated with improved outcomes and decreased mortality. A meta-analysis of eight trials was performed to assess the effect of timing on mortality. A total of *N* = 5324 with a median follow-up of 180 days were included. There was no significant mortality reduction in the early invasive group compared with the delayed invasive group (HR 0.81, 95% CI 0.64–1.03; *p* = 0.0879). However, the prespecified analyses of high-risk patients (elevated cardiac biomarkers at baseline, diabetes, GRACE risk score more than 140, and age older than 75) showed lower mortality in these subgroups [30] irrespective of sex. Despite this, women—who have a higher prevalence of these high-risk comorbidities—are less likely to undergo PCI following NSTEMI, potentially contributing to the worse in-hospital and long-term mortality seen in women [31]. Given higher rates of delayed and atypical presentation in women, use of high-sensitivity troponin assays with gender-specific cut-off may allow for earlier detection and risk stratification of women presenting with NSTEMI and result in higher implementation of guideline-directed medical and procedural treatments [32].

### Stable Ischemic Heart Disease

The role of revascularization plus optimal medical therapy (OMT) over medical therapy alone in managing patients with stable ischemic heart disease (IHD) is debated, with most evidence supporting revascularization for angina relief rather than mortality benefit. Given women’s higher burden of angina [33], this should be considered when deciding medical therapy versus PCI in women with stable IHD. The Clinical Outcomes Utilizing Revascularization and Aggressive druG Evaluation (COURAGE) trial randomized 2287 patients (15% women) to PCI and OMT vs OMT, with a 4.6-year median follow-up time. In a prespecified subset analysis, while there were no sex-specific differences in all-cause death and non-fatal MI, women assigned to PCI had reduced rates of heart failure hospitalization and need for future revascularization [34]. Recent meta-analyses of trials comparing these treatment strategies have shown that revascularization led to reduction in spontaneous MI and greater freedom from angina, reduced cardiac mortality, but no difference in all-cause mortality [35, 36]. More studies are needed to clarify these findings and provide guidance for the management of women with stable IHD.

## Coronary Artery and Atherosclerotic Plaque Characteristics

Advances in intravascular imaging capabilities in the past decade have enhanced our understanding of sex differences in coronary lesion characteristics. While various studies have slightly different findings, likely due to differences in study population and imaging modality, accumulating evidence is suggestive of differential sex-related plaque morphology and atherosclerosis evolution throughout lifespan.

Women tend to have smaller coronary arteries after adjusting for different variables including body surface area and left ventricular mass as well as other demographic and clinical variables [37–39]. Smaller vessel size is associated with lower rates of procedural success and higher rates of in-hospital adverse events following PCI, and increased in-hospital mortality following CABG [40, 41]. Possible explanations for this may be that comparatively smaller plaque burden and thrombus load can result in obstructive disease in smaller vessels. In the long term, smaller vessels may also pose a higher risk for restenosis.

The PROSPECT (Providing Regional Observations to Study Predictors of Events in the Coronary Tree) study described non-culprit (NC) coronary lesions in patients presenting with ACS using multimodality coronary artery imaging (greyscale and radiofrequency intravascular ultrasound, in addition to angiography). Women (25% of study subjects) were older and had higher prevalence of insulin-treated diabetes, hypertension, renal insufficiency, prior congestive heart failure. Despite these comorbid risk factors, compared to men, women had a similar number of culprit lesions and fewer NC lesions which tended to be more focal. While non-culprit plaque burden (PB) and PB > 70% rates were similar among men and women, women had significantly more lesions with MLA < 4.0 mm^2^ after adjusting for BSA and risk factors. More women had at least one NC lesion with MLA ≤ 4 mm^2^ and more had at least 1 high-risk lesion characteristic while more men had at least one lesion with PB > 70% and more frequently had no high-risk lesion characteristics. The primary endpoints (NC-MACE at 1, 2, and 3 years) were similar in men and women. In terms of other NC lesion phenotypes, women had less necrotic core volume, less dense calcium volume, and less fibrous and fibrofatty tissue compared with men. While less necrotic core volume may lower plaque vulnerability for rupture, other high-risk plaque characteristics such as less calcium, smaller MLA, more MLA < 4.0 mm^2^, and significantly less total fibrous volume may counterbalance that effect and explain similar MACE rate observed despite less extensive CAD burden at initial presentation [42]. When compared in age groups ≥ 65 (*n* = 974) vs. < 65 (*n* = 2275), older age group had longer plaques, with greater BP, necrotic core and dense calcium. Men < 65 compared to women < 65 had greater number of fibroatheromas, NCL per patient, larger plaque volumes, and fewer fibrotic plaques [43].

Several other studies, however, did not show significant differences in plaque characteristics among men and women. The OCTAVIA (Optical Coherence Tomography Assessment of Gender Diversity in Primary Angioplasty) study aimed to assess the differences in the underlying mechanism and pathology leading to presentation of STEMI as prior autopsy studies suggested higher incidence of coronary thrombosis associated with nonruptured/eroded plaque in women as opposed to plaque rupture (PR) in men. Among 280 age- and sex-matched subjects (50% women), plaque rupture was similarly present among men and women (50% vs 48.4%), and nonruptured/eroded plaque accounted for 25% of STEMIs in either sex. Similarly, in a study which retrospectively evaluated OCT findings among 211 (23% women) consecutive patients admitted with first STEMI, there was no significant difference in culprit plaque morphology (lipid length, minimum fibrous-cap thickness, and the incidence of PR, thin-cap fibroatheroma, microvessels, macrophages, cholesterol crystals, calcification, and residual thrombus) between men and women [44]. In assessment of stable CAD, retrospective analysis of multimodality intravascular imaging from patients with stable CAD (*N* = 383, woman 30%) showed that plaque morphology—maximum lipid arc, lipid length, lipid volume index, minimum cap thickness, incidence of thin cap fibroatheroma, microvessels, macrophages, and calcification—was similar, as were the plaque characteristics by intravascular US (IVUS). The authors concluded that in the light of similar plaque characteristics, worse outcomes are potentially driven by higher comorbid conditions and differences in primary and secondary preventive measures among women compared to men [45]. In addition to differences in study population, the lack of difference among men and women in these studies may be due to differential progression of coronary atherosclerosis with aging. A large recent study published OCT findings from patients presenting with ACS using data from two registries (Massachusetts General Hospital OCT Registry and Identification of Predictors for Coronary Plaque Erosion in Patients With Acute Coronary Syndrome) stratified by sex and age [46]. Among 1368 patients (20% women), the overall culprit plaque morphology (plaque rupture versus plaque erosion) was similar among men and women. However, significant differences were found among different age groups. In women, prevalence of plaque rupture increased from 26% in those ≤ 50 years to 68% in those > 80 years; this contrasted with men who had a balanced incidence of plaque rupture between 45 and 52% across age groups. In line with an ascending trend in plaque rupture with age, women also had increasing features of plaque vulnerability such as lipid plaque, thin-cap fibroatheroma, and microstructures including macrophages, cholesterol crystals, and calcification.

## Technical Aspect of PCI

### Vascular Access and Access Site Complications

Post-PCI bleeding and vascular access site complications are more common among women and are associated with increased risk of mortality and MACE in this population [26, 47, 48]. This is likely at least partially due to higher prevalence of risk factors such as old age, diabetes, chronic kidney disease, peripheral arterial disease, and smaller vessel size in women. Over the years, advances in procedural technique (smaller sheaths, use of vascular closure devices, ultrasound guidance, radial access) and procedural pharmacotherapy have led to reduced vascular complication rates. [49]

The MATRIX (Minimizing Adverse Hemorrhagic Events by Trans Access Site and Systemic Implementation of AngioX) study randomized 8404 patients to radial or femoral access. Coprimary endpoints were major adverse cardiovascular and cerebrovascular (MACCE) and net adverse clinical events (NACE, defined as MACCE or major bleeding). Women had a higher risk of access site bleeding, severe bleeding, and transfusion. For both coprimary endpoints, the benefit of radial access was relatively greater in women [50]. A meta-analysis of four randomized controlled trials (MATRIX-ACCESS, RIVAL, SAFE-PCI, STEMI-RADIAL) showed in female patients undergoing coronary angiography or intervention, radial access is associated with decreased bleeding, MACCE, and vascular complications, suggesting that radial access should be the preferred approach for women [51]. Despite established improvement in vascular complication rate and bleeding with radial access, it remains underused in women [52], and there is higher rate of crossover (from radial to femoral) in women, likely due to smaller vessel size and tortuosity [53].

With respect to femoral access, various vascular closure devices (VCD) have been developed to improve the safety and efficacy of percutaneous interventions. Instrumental Sealing of ARterial puncture site—CLOSURE device vs manual compression (ISAR-CLOSURE) compared VCDs to manual compression in patients undergoing diagnostic angiography via transfemoral access. VCDs were non inferior to manual compression for the composite end point of vascular complications at 30 days after randomization. Along with that, the incidence of large hematoma was reduced, and efficacy (time to hemostasis) was also significantly improved with VCDs [54]. Similar findings were demonstrated in the CLOSure dEvices Used in everyday Practice (CLOSE-UP) study, which compared the FemoSeal vascular closure device to manual compression [55].

A post-hoc analysis of REGULATE‐PCI (Effect of the REG1 anticoagulation system versus bivalirudin on outcomes after percutaneous coronary intervention) study was undertaken to assess VCD effectiveness and time to hemostasis/ambulation in to focus on subgroups at higher risk of vascular complications. VCD use was associated with a shorter time to hemostasis and time to ambulation; however, bleeding was not different. There were statistically significant 2-way interactions between VCD use and female sex, chronic kidney disease and use of high-potency P2Y12 inhibition with less bleeding observed in the VCD arm among these patients, although future studies are needed to confirm these findings [56].

### Drug-Eluting Stents (DES)

DES are considered the standard of care for patients undergoing PCI. In OCTAVIA study, at 9 months women had similar strut coverage and amount of in-stent neointimal obstruction as men, suggestive of no significant difference in stent performance and vascular response [57]. In a meta-analysis study aimed at assessing safety and efficacy of DES in women, patient-level data for female participants from 26 randomized trials of DES were pooled (*N* = 43,904, 26.3% women). The long-term outcomes according to stent type showed both first and new generation DES were associated with approximately 60% lower rates target-lesion revascularization in women, and lower definite/probable stent thrombosis compared with bare metal stents, as well as lower cumulative incidence of death or myocardial infarction at 3 years [58]. Despite the similar safety and efficacy profile of DES in women compared to men, they are frequently underutilized in women, potentially contributing to worse long-term cardiovascular outcomes in women [28•].

### Intravascular US (IVUS), Optical Coherence Tomography (OCT), and Invasive Ischemia Testing

As previously described, women have smaller heart size and coronary arteries than men, and IVUS measurements show smaller mean vessel area and mean lumen area in women [42]. Despite these differences, there are no sex-specific recommendations for use of OCT or IVUS. But some evidence is suggestive of greater benefit of intravascular imaging for women in optimizing stent expansion and detection and management of stent edge dissection [59]. As discussed previously, given significant prevalence of SCAD in younger women presenting with AMI, judicious use of intravascular imaging can guide diagnosis and management, although extreme care should be taken to avoid iatrogenic dissection.

### Devices Used for Preparation of Calcified Coronary Lesions

Treatment of calcified coronary lesions poses significant challenges and is associated with higher rates of stent thrombosis and target vessel revascularization (TVR). Given older age and higher prevalence of comorbidities compared to men, women present with severely calcified lesions, necessitating lesion preparation by atherectomy or intravascular lithotripsy to allow for optimal stent expansion. Studies comparing outcomes in PCI with rotational atherectomy have demonstrated increased risk of procedural complications and MACE in women compared with men. In a single-center propensity-matched analysis of 765 consecutive patients undergoing RA PCO (37% female) were followed for a median of 4.7 years. The primary endpoint of net adverse cardiac events (all-cause death, myocardial infarction, stroke, target vessel revascularization plus any procedural complication**)** occurred more often in women (15.1 vs. 9.0%; adjusted OR 1.81 95% CI 1.04–3.13; *p* = 0.037), and was driven by an increased risk of procedural complications such as coronary dissection, cardiac tamponade, and significant bleeding [60]. Similarly, in an international multicenter registry of RA PCI, women were more likely to experience in-hospital MACE (defined as cardiovascular death, MI, stroke/TIA, TLR, and CABG), but there was no significant difference in rates of coronary perforation, dissection, slow/low flow or tamponade. In the sex-based analysis of ORBIT II study, a prospective non-randomized multicenter single arm study in the USA evaluating orbital atherectomy system, women had similar rates of in-hospital and 30-day MACE (composite of MI, TLR, and cardiac death) despite higher rates of coronary dissection in women, as well as similar rates of successful stent delivery and < 50% residual stenosis [61]. Disrupt CAD I-IV studies evaluated IVL-facilitated stenting for de novo severely calcified coronary artery lesions in a total of 628 patients (23% women) with stable or unstable angina or silent ischemia. Sex-based analysis of these studies showed no difference in 30-day MACE (composite of cardiac death, MI, or TVR), and similar success rate in stent delivery with ≤ 30% residual in-stent stenosis in women compared to men [62].

### Mechanical Circulatory Support Use in Women

Cardiogenic shock (CS) is a major cause of mortality in patients following STEMI. In addition to early revascularization, shock protocols highlight the importance of early recognition of CS and use of mechanical circulatory support (MCS) devices and invasive hemodynamic monitoring [63]. A retrospective analysis of National Inpatient Sample (NIS) assessing outcomes in STEMI patients with CS (*N* = 159,339, 36.3% women) presenting from 2006 to 2015 showed women were older and had a higher burden of comorbidities and were less likely to have prior PCI or CABG. Women were less likely to undergo right heart catheterization PCI, thrombolysis, CABG, or MCS [64]. The primary outcome of in-hospital mortality was higher among women (range 40 to 45.4%) compared with men (range 30.4 to 34.7%). These disparities were more prominent among women of racial and ethnic minorities.

## Conclusion and Future Directions

Despite significant improvement in our understanding and management of coronary artery disease and its various presentations, there are many important large knowledge gaps in further individualizing current strategies. Majority of the treatment approaches are based on studies in which women comprised 10–20% of study subjects. Given the biological differences discussed previously, a reasonable first step in achieving sustained improvement in outcomes would be better representation of women in trials. In addition, enhancing patient and provider awareness with respect to growing prevalence and worsening outcomes of CAD in women is key to better screening for risk factors—including risk factors specific to women.
